# Nutritional Value Evaluation and Processing Technology of Feed and Nutrition Regulation Measures for Ruminants

**DOI:** 10.3390/ani14213153

**Published:** 2024-11-02

**Authors:** Dingfu Xiao, Tiantian Meng

**Affiliations:** 1 Yuelushan Laboratory, College of Animal Science and Technology, Hunan Agricultural University, Changsha 410128, China; xiaodingfu2001@hunau.edu.cn; 2 College of Life Science, Xinyang Normal University, Xinyang 464000, China

## 1. Introduction 

In the context of modern livestock farming, particularly in the ruminant industry, feed is a crucial factor that affects both the efficiency of production and animal health. Over the last decade, rising feed costs, driven by factors such as increased demand, raw material shortages, and global crises such as COVID-19, have posed significant challenges to the cattle and sheep farming industries. Feed expenses make up the largest part of production costs, and the price of essential feed ingredients such as corn has surged, placing increased economic pressure on farmers [1]. Thus, understanding the nutritional value of alternative feed sources and improving feed processing technologies is essential in order to enhance the productivity, product quality, and sustainability of livestock.

High-concentrate diets are often rich in carbohydrates. When ruminants consume these diets, volatile fatty acids (VFAs) such as acetic, propionic, and butyric acids are produced in the rumen through microbial fermentation. However, the absorption capacity of the rumen wall is limited, leading to the accumulation of VFAs, which lowers the pH of the rumen and triggers subacute ruminal acidosis (SARA) [2]. The prolonged consumption of high-concentrate diets increases the risk of SARA, disrupting rumen metabolism and impairing normal rumen function, leading to a reduction in production. Therefore, it is crucial to mitigate SARA through strategies such as the bioconversion of unconventional feeds and the use of plant extracts to prevent the negative effects associated with high-concentrate diets.

## 2. Feed Ingredients and Nutritional Value

The nutritional value of feed varies significantly depending on its source, variety, and processing methods. The quality of forage, including its fiber content, digestibility, and nutrient availability, plays a critical role in the overall health and productivity of ruminants [3]. High-quality forage promotes enhanced digestion and nutrient absorption, ensuring that livestock can maintain optimal health and performance levels. On the other hand, poor-quality feed leads to digestive problems and reduced productivity, which can result in significant economic losses [4]. In contrast, feeding overly mature grasses to ruminants increases the production of methane, which is strongly correlated with the carbon-to-nitrogen ratio in grasses [5,6]. Additionally, the nutrient content of different forages can affect the production of methane post-feeding [7]. Leguminous forages produce less methane than grasses, as the condensed tannins in legumes significantly impact the degradation of fiber and dry matter [8,9,10,11]. Not only does the quality of forage affect methane emissions, but different processing methods can also influence the production of methane in ruminants. For example, treatments such as ammonization, crushing, pelletizing, and ensiling have been shown to reduce methane emissions to varying degrees [12]. This reduction is likely due to an increase in the levels of non-fibrous carbohydrate (NFC) after ensiling, which lowers the NDF content and promotes the production of propionic acid in the rumen while reducing acetic acid; thus, methane synthesis is decreased [13,14]. Furthermore, high-concentrate diets, which are commonly used in intensive ruminant production, are rich in carbohydrates. Therefore, evaluating the nutritional value of feed ingredients and formulating balanced diets that prevent digestive disturbances are crucial to ensuring the well-being of ruminants.

## 3. Feed Processing Technology

The processing of feed plays a pivotal role in maintaining or even enhancing its nutritional quality. Technologies such as ensiling, pelleting, and steam-flaking can help preserve nutrients and enhance the efficiency of feed [15]. For instance, ensiling is a method used to preserve forage crops via fermentation, which not only extends the shelf life of feed but also enhances its digestibility by breaking down fiber components [16]. Advances in feed processing have enabled the enhanced utilization of alternative feed ingredients, thus mitigating the high costs associated with conventional feed materials.

Moreover, bioconversion techniques, including the microbial fermentation of unconventional feedstuffs, have gained attention due to their ability to enhance the value of feed while reducing dependence on traditional, high-cost ingredients [17]. This method can transform low-nutrient materials into more digestible and nutritionally valuable feed through the action of microbes, making it an attractive option for sustainable livestock farming.

## 4. Nutrition Regulation Measures

To address the challenges posed by high-concentrate diets, various nutritional regulation strategies have been explored. One approach involves the use of plant extracts and essential oils as natural additives to prevent the negative effects of rumen acidosis. These additives have been shown to modulate rumen fermentation patterns, promoting the production of beneficial VFAs while reducing methane emissions [18]. This not only enhances the health of the rumen, but also contributes to environmental sustainability by mitigating the greenhouse gas emitted from livestock [19].

Another promising nutritional regulation strategy is the integration of precision feeding techniques. This involves the use of real-time monitoring systems to adjust the composition and delivery of feed based on the requirements of individual animals [20]. Precision feeding can optimize the utilization of nutrients, improve the performance of animals, and reduce waste, leading to a more efficient and sustainable production system.

### 4.1. Unconventional Feeds for Ruminants

Unconventional feed resources possess unique nutritional qualities, but are associated with various challenges; these include processing difficulties, high transportation costs, susceptibility to deterioration, poor palatability, the presence of anti-nutritional factors, strong seasonality, and limited geographical availability. To maximize their benefits, these resources must be developed rationally and used in a scientifically sound manner. For example, the dietary supplementation of DHA-rich microalgae improved the antioxidant status, increased the deposition of DHA and enhanced the characteristic flavor of beef [21]. Silva found that partially substituting corn silage with whole soybean silage or black oat silage reduced the intake of feed while increasing rumination and chewing activities in cows, without compromising growth performance [22]. Similarly, Zhang et al. reported that sweet straw and wheat straw could serve as more affordable alternatives to whole-plant corn silage (WPCS) for fattening beef cattle, without significantly diminishing the economic returns [23]. Additionally, the inclusion of whole pomegranate byproducts in sheep diets, replacing traditional grain feed, improved the antioxidant capacity of the meat, likely due to the presence of vitamin E in the pomegranate byproducts [24].

### 4.2. Applied Plant Extract

A recent study using rumen simulation technology evaluated the effects of chito-oligosaccharides (COS) (0.02%, 0.04%, 0.08%) on the diets of beef cattle. The results showed that COS increased the disappearance rates of dry matter DM, crude protein (CP), ether extract, and acid detergent fiber, while raising the pH, microbial CP, and concentrations of propionate and butyrate. It also reduced the production of gas, NH3-N, the A/P ratio, and the acetate, isobutyrate, valerate, and protozoa counts, shifting fermentation from an acetate to a propionate mode. COS did not change the overall diversity of the microbiota but increased the relative abundance of *Methanosphaera*, *Ruminococcus*, *Endomycobium*, and *Eubacterium* [25].

In vitro experiments were conducted by adding mixtures of Firmwood tannin and chestnut tannin to forage and hay. The addition of these tannin mixtures to hay significantly reduced the acetic acid-to-propionic acid ratio; after 24 h, the CO_2_/CH_4_ ratio increased notably with 30 g/kg of mixed tannins. While the addition of mixed tannins to hay significantly lowered the production of ammonia, no such effect was observed in forage. Mixed tannins also protected against the degradation of protein in the rumen, particularly under low-protein conditions; this was possibly due to the expansion of the binding spectrum [26]. Furthermore, the addition of 1% and 2% Neolamarckia cadamba leaf extracts significantly reduced the production of CH_4_ and increased the digestibility of dry matter, although it did not affect the concentrations of VFAs. While the microbial community of the rumen was largely unaffected, the extracts significantly decreased the relative abundance of *Methanobacterium*. This reduction in methane-producing bacteria was positively correlated with a reduction in the production of CH_4_ in the rumen [27].

## 5. Conclusions

The rising cost of feed and the challenges associated with maintaining ruminant health in intensive production systems necessitate a comprehensive approach to evaluating the nutritional value of feed and processing technologies. By leveraging bioconversion methods, improving the quality of feed, and implementing effective nutrition regulation measures, the ruminant industry can achieve enhanced productivity and sustainability. Further research is needed to develop innovative feed solutions and nutritional strategies that not only enhance animal performance but also reduce the environmental impact of livestock farming.
